# Neurocognitive Correlates of Cerebellar Volumetric Alterations in Youth with Pediatric Bipolar Spectrum Disorders and Bipolar Offspring

**DOI:** 10.2174/1570159X21666221014120332

**Published:** 2023-05-12

**Authors:** Kirti Saxena, Alessio Simonetti, Christopher D. Verrico, Delfina Janiri, Marco Di Nicola, Antonello Catinari, Sherin Kurian, Johanna Saxena, Benson Mwangi, Jair C. Soares

**Affiliations:** 1 Menninger Department of Psychiatry and Behavioral Sciences, Baylor College of Medicine, Houston, Texas, TX, USA;; 2 Department of Psychiatry, Texas Children’s Hospital, Houston, Texas, TX, USA;; 3 Department of Neuroscience, Section of Psychiatry, Fondazione Policlinico Universitario “Agostino Gemelli” IRCCS, Rome, Italy;; 4 Department of Neurology and Psychiatry, Sapienza University of Rome, Rome, Italy;; 5 Department of Neuroscience, Section of Psychiatry, Università Cattolica del Sacro Cuore, Rome, Italy;; 6 Department of Psychiatry and Behavioral Sciences, University of Texas Health Science Center, Houston, Texas, TX, USA

**Keywords:** Pediatric bipolar disorder, cerebellum, emotion processing, cognition, offspring, magnetic resonance imaging

## Abstract

**Background:**

Emerging evidence points towards the involvement of the cerebellum in the processing of emotions and pathophysiology of mood disorders. However, cerebellar and related cognitive alterations in youth with pediatric bipolar disorder (PBD) and those at high risk to develop the disorder, such as bipolar offspring (BD-OFF) are not clearly defined.

**Objective:**

To investigate cerebellar gray and white matter volumes, cognition, and their relationship in youth with PBD and BD-OFF.

**Methods:**

Thirty youth (7 to 17 years, inclusive) with PBD, 30 BD-OFF and 40 healthy controls (HC) were recruited. Study participants underwent a computer-based cognitive battery assessing affective processing, executive function, attention, psychomotor speed, and learning. Three-tesla MRI scan was performed to assess cerebellar white and gray matter volumes. Cerebellar segmentation was performed with FreeSurfer. Statistical analyses include between-group differences in cognitive domains, cerebellar gray, and white matter volumes. Relationships between cerebellar volumes and cognitive domains were examined.

**Results:**

Youth with PBD showed greater cerebellar gray matter volumes than both BD-OFF and HC, whereas no differences were present between BD-OFF and HC. Both youth with PBD and BD-OFF showed altered processing of negative emotions and a bias towards positive emotions. In youth with PBD and BD-OFF, greater impairment in the processing of emotions correlated with greater cerebellar gray matter volumes.

**Conclusion:**

The present findings corroborate hypotheses on cerebellar involvement in the processing of emotions and the pathophysiology of PBD. The presence of cerebellar dysfunction in BD-OFF is unclear.

## 1 INTRODUCTION

Pediatric bipolar disorder (PBD) is marked by episodes of mania or hypomania and depression which are characterized by rapid mood changes, mixed mood-states, and chronic morbidity [1-3]. Also, PBD is associated with alterations in several cognitive domains, such as attention [4, 5], psychomotor speed [6], executive functions [4, 7-10] and processing of emotions [11, 12]. Alterations in emotionally-valenced stimuli recognition and processing have been addressed as a pivotal mechanism of illness onset and progression [13]. Additionally, altered emotion processing has been found in youth at high risk to develop PBD, such as bipolar offspring (BD-OFF) [14], thus indicating a possible marker of disease predisposition. Therefore, the assessment of cognitive alterations in youth with PBD and at-risk populations is of significance.

Notably, the study of anatomical underpinnings of these cognitive alterations might be a viable tool to identify reliable neuromarkers of the progression and predisposition to mood disorders early on. In the last decades, structural magnetic resonance imaging (sMRI) studies revealed several volumetric alterations in youth with PBD and BD-OFF in areas related to the previously mentioned cognitive domains [11, 15-17]. However, research on neuromarkers and their neurocognitive correlates focused mainly on prefrontal or fronto-limbic areas [18-20], whereas less attention has been given to areas external to the fronto-limbic circuitry. Indeed, the cerebellum has gained increasing attention for its involvement in emotional processes [21]. The cerebellum contains almost 80% of the brain’s neurons and plays a primary role in balance, posture, coordination, and speech [22, 23]. However, the presence of disinhibition, overfamiliarity, flamboyant and impulsive actions, flippant comments, and inappropriate behaviour in individuals with cerebellar lesions led to the hypothesis of the involvement of cerebellar circuits in the pathophysiology of emotional processing, and, consequently, of mood disorders [21, 24, 25]. The increasing evidence of an underlying cerebellar dysfunction in cognitive domains commonly impaired in PBD [26-29] has provided additional support to the hypothesis of the role of cerebellar dysfunction in such a population. sMRI findings on gray matter volumetric alterations in youth with PBD and BD-OFF are few and results are inconsistent. Nevertheless, there is a weak majority of studies reporting smaller gray matter volumes in youth with PBD and BD-OFF [30-34], even though greater volumes have also been found [35, 36]. On the other hand, studies investigating white matter cerebellar volumetric alterations are scarce and reported the absence of alterations [37, 38]. Therefore, the presence of cerebellar volumetric alterations in youth with PBD and BD-OFF warrants further clarification. Additionally, there is a need to investigate the possible cognitive correlates of cerebellar dysfunction in youth with PBD and BD-OFF.

This study assessed cerebellar gray and white matter volumes, and cognitive functions in youth with PBD, BD-OFF, and healthy controls (HC). Since there is robust evidence documenting the presence of alterations in executive function and emotion processing in PBD, we expected PBD to show alterations in these two domains compared to HC [11, 12, 39]. Since altered emotion recognition and processing have been hypothesized to be a core feature of PBD and possibly a vulnerability biomarker, we expected to find alterations in this domain in both PBD and BD-OFF in comparison to HC. In accordance with the majority of literature’s findings, smaller cerebellar gray matter volumes were expected. Given increasing evidence regarding the relationship between cerebellar dysfunction and emotion processing, we expected that in both youths with PBD and BD-OFF, smaller cerebellar volumes would correlate with greater impairment in emotional processing.

## 2 MATERIALS AND METHODS

This study was approved by the Institutional Review Boards (IRB) at the Baylor College of Medicine in Houston, TX and the University of Texas, Houston, TX (IRB number: H-50361). The parent or legal guardian of each study participant was informed of the procedures and potential risks involved in this study before they provided written consent for their child/adolescent to participate. Each study participant verbally assented to their participation in this study.

### 2.1 Participants

The study participants were 100 in total; 30 in the PBD group, 30 in the BD-OFF group, and 40 in the HC group (see Table **1** for demographics). Study participants included females and males, 7-17 years old. Suitability for MRI scanning was also required for all the youths to be included. Youth with PBD also required (i) A diagnosis of PBD; (ii) the absence of an eating disorder, ADHD and anxiety disorders without comorbid PBD. BD-OFF also required: (i) at least a first-degree relative with a diagnosis of bipolar disorder (BD); (ii) absence of a diagnosis of a mood disorder (*i.e*. major depressive disorder, bipolar disorder type I, II or not otherwise specified (NOS), cyclothymic disorder, dysthymic disorder). HC also required: the absence of psychiatric illness, and the absence of any psychiatric illness in first-degree relatives. Exclusion criteria for the whole sample include (i) comorbid substance use disorder; (ii) intellectual disability; (iii) comorbid autism spectrum disorder; (iv) severe neurological conditions.

### 2.2 Psychiatric Assessments

Standardized diagnostic interviews using (the Mini International Neuropsychiatric Interview version 7.0.1 for Diagnostic and Statistical Manual of Mental Disorders, version 5 (DSM-5) (MINI) [40] were conducted with each study participant to confirm a diagnosis of PBD. The MINI was also conducted with the biological parent of the bipolar offspring to confirm the parent’s diagnosis of BD. The Wechsler Abbreviated Scale of Intelligence - II (WASI-II) [41] was administered to determine age- and sex-corrected general intelligence (composite IQ score). Additionally, standardized rating scales were utilized to assess the severity of manic (the Young Mania Rating Scale - YMRS) [42] and depressive (the Children's Depression Rating Scale - CDRS) [43] symptoms. YMRS is an eleven-item, clinician-administered rating scale used to measure the severity of manic symptoms in children and adolescents between the ages of 5 and 17, during the past 7 days. The range of scores is 0-60. A score of 12 or above is considered reflective of hypomania and mania. CDRS is a widely used, clinician-administered instrument which assesses the severity of depressive symptoms in children/adolescents. It is a 17-item scale with items rated between 1 (=no difficulties) and 5 or 1 and 7 (=clinically significant difficulties), adding up to a total score between 17 to 113. On the CDRS, a score of 40 and above is clinically significant. These scales were completed for all study participants at baseline.

### 2.3 Cognitive Testing

Study participants completed a series of tasks within the Cambridge Neuropsychological Test Automated Battery (CANTAB, https://www.cambridgecognition.com/cantab/), notably the Affective Go/no-go task (AGN), the Cambridge Gambling Task (CGT), the Stockings of Cambridge task (SOC), Match to Sample Visual Search task (MTS), the Big/Little Circle task (BLC). Cognitive domains assessed were affective processing, executive functions, attention and psychomotor speed, and learning.

#### 2.3.1 Affective Processing

Affective processing was assessed with the AGN. In this task, participants are presented with positive words (*e.g*., “laughter”, “happiness”, “joyful”) and negative words (*e.g*., “gloom”, “ashamed”, “sadness”, *etc*.). Before each round, the participant is instructed to press a button on the target which is either the positive or negative words. If asked to press on a positive word and the participant presses on a negative word, this is considered a commission error (CE) for negative words. On the other hand, if the participant is asked to press on a positive word and the participant does not do so, then this is considered an omission error for positive words. Outcome measures are reaction times (AGN-RT) for positive (AGN-RT-positive) and negative (AGN-RT-negative) words, CE for positive (AGN-CE-positive) and negative (AGN-CE-negative) words and omissions for positive (AGN-omissions-positive) and negative (AGN-omissions-negative) words.

#### 2.3.2 Executive Functions

Executive functions were assessed using the Cambridge Gambling task (CGT) and the Stockings of Cambridge task (SOC).

The CGT is specifically tailored to assess decision-making and risk-taking behavior. Study participants are presented with a row of ten boxes across the top of the screen: some are red, and some are blue. They must use the 'Red' and 'Blue' buttons at the bottom of the screen to choose the box color in which they think the token is hidden. The study participants start with 100 points and select a proportion of these points to bet on their decision. A circle in the center of the screen displays the current bet value, which will either incrementally increase or decrease (depending on the task variant selected). Participants press this button when it shows the proportion of their score they would like to bet. These points will either be added to or taken from their total score, depending on their decision and where the token is hidden. Outcomes are delay aversion (CGT-delay aversion), deliberation time (CGT-deliberation time), proportion bet (CGT-proportion bet), quality of decision making (CGT-quality of decision making), risk adjustment (CGT-risk adjustment), risk taking (CGT-risk taking).

The SOC is an executive function task designed to measure spatial planning and strategic reasoning. It presents the study participant with balls in stockings in a specific pattern on the top and bottom of the screen, and the study participant is instructed to manipulate the balls on the bottom of the screen to match the top. Outcome measures include the number of problems solved in minimum moves (SOC-moves).

#### 2.3.3 Attention and Psychomotor Speed

Attention and psychomotor speed were evaluated with the Match to Sample Visual Search task (MTS). In this task, the study participant is shown a complex visual pattern in the middle of the screen. After a brief delay, a varying number of similar patterns are shown in a circle of boxes around the edge of the screen. Only one of these patterns matches the pattern in the center of the screen, and the participant must indicate which it is by selecting it. The outcome measure for this task is accuracy (MTS-% of correct choices).

#### 2.3.4 Learning

Learning has been assessed with the Big/Little Circle task (BLC). In this task, two circles are displayed. The study participants must first select the smaller of the two circles, then, after 20 trials, select the larger circle for a further 20 trials. The outcome is the study participant’s ability to select the correct circle (BLC-% of correct selection).

### 2.4 Imaging

Study participants underwent a structural MRI scan to determine cerebellar gray and white matter volumes. Neuroimaging scans were acquired using a research dedicated Philips Ingenia 3T MRI system. T1-weighted neuroimaging scans were acquired for anatomical analysis with the following scanner parameters - Repetition time (TR) = 8.1 ms, echo time (TE) = 3.68 ms, field of view (FOV)= 256 mm × 180 mm voxel size = 1 mm × 1 mm × 1 mm. Cerebellar volumes were segmented and quantified using FreeSurfer suite version 6.0 (38). FreeSurfer is a fully automated software that performs motion correction, intensity normalization, automated topology correction, and atlas-based cortical/subcortical segmentation and labeling of MRI images. Cerebellar gray and white matter were corrected for intra-cranial volume following the proportion method.

### 2.5 Statistical Analyses

Statistical analyses for this study were performed with IBM SPSS Statistics 22.0 for MAC (IBM Corporation). FreeSurfer was used to analyze the data.

#### 2.5.1 Demographic and Clinical Characteristics

One-way factorial analyses of variance (ANOVAs) for continuous variables (*i.e*., age, IQ, CDRS and YMRS scores), and chi-square tests for nominal variables (*i.e*., gender, race/ethnicity, comorbidities, current pharmacological treatments) were performed to assess for group differences in demographic and clinical characteristics (Table **1**).

#### 2.5.2 Cerebellar Gray and White Matter Volumes, Cognitive Testing

One-way factorial analyses of covariance (ANCOVAs) were performed to investigate between-group differences in cognition, cerebellar gray, and white matter volumes. In each ANCOVA, the three study groups (*i.e*., PBD, BD-OFF, HC) were used as independent variables, whereas CANTAB tests scores, and left and right cerebellar gray and white matter volumes were used as dependent variables. Age, sex, and IQ were used as variables of no interest. Correction for multiple testing (*p* = .05/4 = .0.0125 for cerebellar gray and white matter volumes; *p* = .05/16 = .0031 for CANTAB tests scores) was performed. ANCOVAs were followed by Bonferroni *post hoc* tests to examine between-group differences with *p* < .05 as the level of significance. Cohen’s *d* was used as a measure of effect size. To further minimize the likelihood of type-I error, univariate ANCOVAs were preceded by overall multivariate analyses of covariance (MANCOVA), in which CANTAB tests scores, left and right gray and white matter volumes were used as dependent variables, whereas groups were used as an independent variable. Age, gender, and IQ were used as covariates. Since only AGN and the CGT task embedded multiple outcomes, MANCOVAs were limited to these two tasks.

#### 2.5.3 Relationships between Cognition and Cerebellar Volumes

General linear models (GLMs) were used to compare relationships between gray and white matter cerebellar volumes and cognitive performances among groups. Specifically, each CANTAB test score was used as a response variable, left and right gray and white matter volumes were used as predictors and, the study group was used as a covariate. Age, gender, and IQ were entered as variables of no interest. A significant interaction effect was further investigated with multiple univariate hierarchical regressions for each group (youth with PBD, BD-OFF and HC) separately, controlling for the effect of age, gender, and IQ. Pearson’s chi square analyses were then used to assess the direction of the associations found.

## 3 RESULTS

### 3.1 Demographic and Clinical Characteristics

The study sample included 30 study participants with PBD, 30 BD-OFF, and 40 HC. As shown in Table **1**, demographic differences were non-significant among groups. Regarding the severity of manic symptoms, youth with PBD were mildly hypomanic, so they were not severely clinically impaired on mania scores. Similarly, the youth with PBD were sub-clinically depressed, as their depression scores were lower than the clinical cut-off of 40 on the CDRS scale. As regards psychopharmacological treatments, subjects with PBD assumed more antidepressants (ADs), antipsychotics (APs) and mood stabilizers (MSs) than BD-OFF.

### 3.2 Cognition

MANCOVAs revealed a global main effect for AGN (Wilks’ Lambda = .72; F = 2.34; df = 12; *p* = .009) and CGT (Wilks’ Lambda = .77; F = 1.92; df = 12; *p* = .035) tasks.

Multiple one-way ANCOVAs showed significant differences in the AGN-CE-positive, AGN-omission-positive, in CGT-deliberation times and SOC-moves. Differences in AGN-omission-negative only approached significance. Post-hoc analyses showed that both youth with PBD and BD-OFF showed more AGN-CE-positive than HC. Youth with PBD showed more AGN-omissions-positive than both BD-OFF and HC. As regards the CGT, youth with PBD showed longer deliberation times than both BD-OFF and HC. No other between-group differences emerged. Results are shown in Table **2**.

### 3.3 Cerebellar Gray and White Matter

MANCOVAs revealed a main global effect for the right (Wilks’ Lambda = .58; F = 33.55; df = 2; *p* < 001) and the left (Wilks’ Lambda = .59; F = 36.88; df = 2; p = *p* < .001) cerebellar volumes. One-way ANCOVAs revealed significant differences in both left and right gray and white matter cerebellar volumes amongst groups. Post-hoc analyses revealed that youth with PBD showed significantly greater gray matter cerebellar volumes bilaterally than both BD-OFF and HC. The latter two groups did not show significant differences. No differences emerged amongst groups in left and right white matter volumes. Results are shown in Table **3**.

### 3.4 Relationships between Cognition and Cerebellar Volumes

**Fig. (1):**
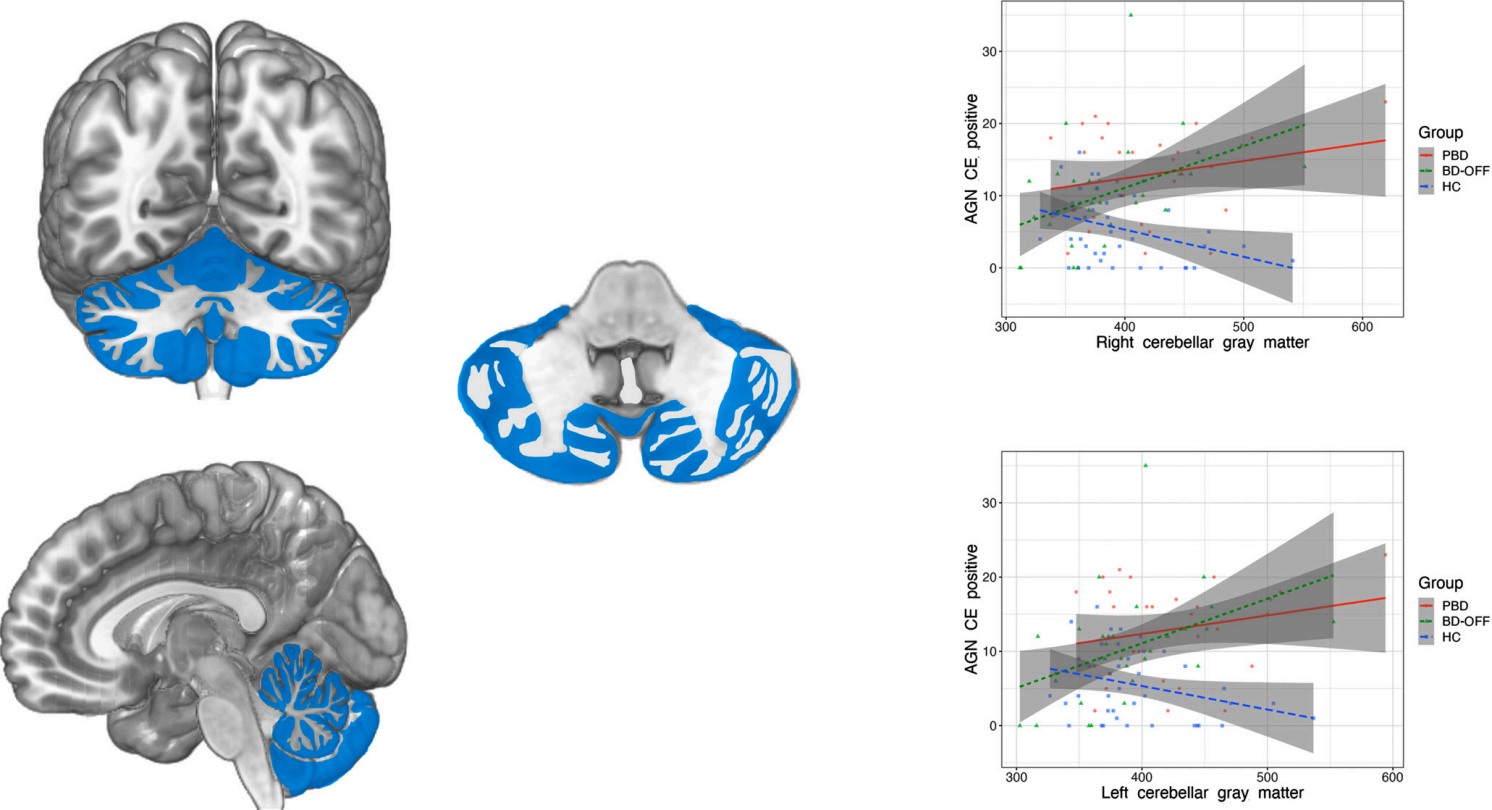
Correlation between left and right cerebellar gray matter volumes and AGN-CE-positive in youth with PBD, BD-OFF, and HC. **Abbreviations**: PBD, youth with pediatric bipolar disorder; BD-OFF, offspring of subject with bipolar disorder; HC, healthy controls. AGN, affective Go/no-go task; CE, errors.

As differences amongst groups were limited to the left and right cerebellar gray matter volumes, AGN-CE-positive and AGN-omission-positive, CGT-deliberation times, GLMs were limited to these variables. Significant groups by right- and left- cerebellar gray matter volume interactions were found for AGN-CE positive (F = 6.35; *p* = .001; F = 5.50; *p* = .002, respectively). Multiple hierarchical regression analyses identified a significant relationship between the left and right cerebellar gray matter and AGN-CE-positive in both youths with PBD and BD-OFF. In HC, regression only approached significance. Partial correlation analyses showed that bilateral cerebellar gray matter volumes were positively correlated with the number of CE-positive in both youths with PBD and BD-OFF. Results are shown in Table **4** and Fig. **1**.

### 3.5 Effect of Possible Confounding Variables

Since the duration of illness and medication have been proven to affect brain volumes [44, 45], the confounding effect of such variables on gray and white matter cerebellar volumes were investigated. As regards the effect of the duration of illness, multiple linear regressions were performed in youth with PBD only. In each regression, the duration of illness was used as a predictor, and cerebellar volumes were used as outcome variables. Results showed no significant correlations among the aforementioned variables (F = .22, *p* = .641 for left cerebellar white matter; F = .39, *p* = .534 for right white matter; F = 1.67, *p* = .287 for left cerebellar gray matter; F = 1.26, *p* = .269 for right cerebellar gray matter). As regards the possible effect of medications multiple two-way ANOVAs were performed in youth with PBD and BD-OFF only. In each ANOVA, gray and white matter cerebellar volumes were dependent variables, whereas groups (PBD and BD-OFF) and the presence or absence of AD, AP, MS, and stimulants were independent variables. The small number of subjects receiving benzodiazepines (BDZ) impeded performing a reliable statistic. Therefore, between-group differences regarding cerebellar volumes were recalculated after subtracting the subjects under BDZ (2 for youth with PBD, 3 for BD-OFF). No interaction effects were found (F = .84, p = .438 for AD; F = .06, p = .806 for AP; F = .03, *p* = .870 for MS; F = 1.35; *p* = .312 for stimulant). After subtracting subjects with BDZ, results remained unchanged (F = 2.23, *p* = .112 for left cerebellar white matter; F = 2.54, p = .08 for right cerebellar white matter; F = 4.88, p = .10 for left cerebellar gray matter; F = 4.38, p = .15 for right cerebellar gray matter). Therefore, the effect of the duration of illness and medications was not further investigated.

## 4 DISCUSSION

Results can be summarized as follows: 1) youth with PBD had worse performances in affective processing executive function than HC; 2) youth with PBD had larger cerebellar gray matter volumes than both BD-OFF and HC; 3) BD-OFF had worse performances in affective processing than HC; 4) cerebellar gray matter volumes correlated with impairment in affective processing in both youth with PBD and BD-OFF.

Results from this study confirm previous findings reporting alterations in affective processing [46, 47] and executive functions [4, 47, 48] in youth with PBD. Results on cerebellar volumes differ from what was expected: cerebellar gray matter volumes were larger in youth with PBD as compared with those of BD-OFF and HC. These findings are in line with those of Adler *et al.* 2007 [49], whereas, they contrast with those of Demirgören *et al.* 2019 [30], Moberget *et al.* 2019 [50] and James *et al.* 2011 [31]. Differences in methodology might explain discrepancies among studies. First, Moberget included youth with different diagnoses, whereas James *et al.* included youth with PBD and psychotic features. Secondly, sample sizes in the studies of Demigroen *et al.* and James *et al.* are small. Thirdly, all the studies used different MRI segmentation techniques. On the other hand, neurodevelopmental trajectories should be considered while interpreting our findings. Gray matter volume follows a U-shaped trajectory over time and reaches its peak across puberty [51]. Gray matter peak varies depending on the brain areas involved, with the frontal lobes peaking at age 11 and the cerebellum at 13 [50]. Then, synaptic pruning, *i.e*., the microglia-mediated, targeted elimination of functional synapses, takes place with the aim of increasing the efficacy of neural networks. Given the present study’s mean age, *i.e*. 13 years-old, the present findings suggest poor synaptic pruning in youth with PBD. Synaptic pruning has not been confirmed empirically in PBD [52], however, results from this study corroborate those of Patel and colleagues [53], who showed an increased level of N-acetyl aspartate, an indirect marker of synaptogenesis in adolescents with BD, and are in line with those of Seredenina *et al.* [54] who found reduced expression of NADPH oxidase - a marker of microglial activity - in postmortem brains of individuals with BD. Delayed maturational cerebellar patterns in youth with PBD might be an additional hypothesis to explain the aforementioned findings. Deceleration of brain aging structural trajectories has been already noticed in young individuals with recent-onset PBD [55, 56] and has been proposed as a predisposing factor for emotion control network disruption [57]. This process can further lead to accelerated brain aging when youth with PBD age [58-60].

The positive relationship between cerebellar gray matter volumes and AGN-CE-positive corroborates findings on the cerebellar involvement in affective regulation [22]. The cerebellum has broad connections with areas involved in emotional processing, such as the amygdala, the hippocampus, the hypothalamus, the insula, and the anterior cingulate cortex [61-63]. The role of the cerebellum includes emotion recognition and handling of strong incoming stimuli that are predictive of a change in emotional demands or context, necessitating the rapid synchronization of emotional information strands to optimize response [64]. Multiple evidence highlight that this function is specific for negative emotions [65]. To this extent, transcranial cerebellar cortex stimulation selectively enhances facial anger and sadness [66], whereas functional MRI studies report that exposure to negatively-valenced stimuli strongly activates the cerebellum, whereas positive did not [67]. Our findings of a specific correlation between gray matter volumes and AGN-CE-positive might suggest that poor cerebellar pruning/maturation might turn into negative emotion recognition and processing. Such dysfunction might lead to a propensity towards recognition of negative emotions as positive.

BD-OFF showed greater AGN-CE-positive, *i.e*., positive bias, than HC, thus corroborating previous evidence of altered negative emotion processing in BD-OFF [68]. Positive bias has been related to the predisposition to mania [69] and has been recently proposed as an endophenotype of BD [70]. The correlation found in either youth with PBD and BD-OFF of gray matter cerebellar volumes and commission errors for positive stimuli suggests that such endophenotype might depend, at least in part, on cerebellar dysfunction. However, different from individuals with BD, BD-OFF showed no cerebellar volumetric differences from HC. This finding is in contrast with those who identified smaller volumes in BD-OFF [31-33] and with Lin *et al.* [35], who found larger cerebellar volumes in the BD-OFF as compared with HC. However, it is worth mentioning that these studies focused on adults or included mixed samples of adolescents/young adults. When more strict criteria were applied [71], results overlap with the present study. A possible explanation of the discrepancies might rely on the poor capacity of structural MRI to detect subtle alterations in brain structure, as demonstrated by the non-linear relationship between structural MRI and brain functions [72]. Alternatively, the present segmentation method does not allow the determination of specific regional anatomic cerebellar alteration in BD-OFF. Findings from Frangou [33] and Kempton *et al.* [32] demonstrated that cerebellar alterations were limited to the left cerebellar vermis, whereas the present study’s segmentation method does not allow the determination of volumetric measurements in cerebellar subregions. Consequently, volumetric alterations in BD-OFF might be subtle and would not be detected by the segmentation used, even though they lead to altered cerebellar-related functions, such as emotion recognition. After considering the limitations of the segmentation method used, it might be hypothesized that cerebellar alteration might be subtle before the illness's onset. However, it would be sufficient to cause relevant alterations in functions, *i.e*., poor emotion recognition and processing. After the illness onset, cerebellar alterations would be generalized enough to be detected by segmentation techniques.

## 5 LIMITATIONS

The small sample size in this study does not allow generalizability of the results found, and larger sample sizes are needed to confirm the present findings. Furthermore, a longitudinal design is needed to clarify hypotheses on developmental structural-cognitive trajectories in youth with PBD and BD-OFF. Additionally, results are hampered by non-specific methods assessing white matter alterations. More sophisticated techniques, such as tractography, together with more specific indices of white matter fibers alterations, such as fractional anisotropy and main diffusivity, are needed to better define white matter alterations. As previously stated, more sophisticated segmentation methods are also necessary to localize cerebellar gray matter alterations in these two populations. Analyses performed on the possible effect of medications were limited only to drug classes. Additional studies are needed to investigate the effects of specific medications, which have been proven to affect behavior [73, 74], and brain volumes [45]. Other possible variables that can affect the present findings, such as characteristics of mood episodes and states [75] and cyclicity [76, 77] should be evaluated.

## CONCLUSION

The present findings corroborate hypotheses on cerebellar involvement in mechanisms related to emotional control in PBD. Alterations in processes partially subserved by cerebellum and present in youth with PBD are also present in BD-OFF, even though they are not corroborated by structural alterations. Additional studies, with larger sample sizes and more sophisticated MRI techniques are needed to clarify the relationship between cerebellar alteration and cognition in youth with PBD and BD-OFF.

## Figures and Tables

**Table 1:** Demographical and clinical characteristics of 30 youth with PBD, 30 BD-OFF and 40 HC.

			**PBD ** **(N = 30)**	**BD-OFF ** **(N = 30)**	**HC ** **(N = 40)**	***F* or χ^2^**	***df***	***p-value***
** Demographics**								
Age (y), mean ± SD			12.14 ± 3.428	11.19 ± 3.020	12.91 ± 2.776	2.71	2	.07
Female, n (%)			14 (46.7)	15 (50.0)	18 (45.0)	.17	2	.92
Employed, n (%)			10 (41.7)	23 (54.8)	21 (61.8)	2.31	2	.32
** Race**								
Asian, n (%)			0 (0.0)	0 (0.0)	3 (7.5)	11.24	2	.08
African-American, n (%)			3 (10.0)	9 (30.0)	9 (22.5)	-	-	-
Caucasian, n (%)			25 (83.3)	16 (53.3)	23(57.5)	-	-	-
Mixed/Other, n (%)			2 (6.7)	5 (16.7)	5 (12.5)	-	-	-
** Ethnicity**								
Hispanic, n (%)			6 (20.0)	12 (40.0)	10 (25.0)	3.27	2	.20
I.Q., mean ± SD			97.50 ± 16.660	95.13 ± 13.019	100.67 ± 11.902	1.42	2	.25
** Clinical Charachteristics**								
Year ill (y), mean ± SD			8.21 ± 5.395	-	-	-	-	
CDRS-R, mean ± (SD)			34.13 ± 13.400	22.90 ± 7.039	17.82 ± 1.852	33.18	2	**< .01**
YMRS, mean ± (SD)			12.83 ± 10.926	3.10 ± 3.595	.88 ± 1.620	32.36	2	**< .01**
**PBD Type**								
Type 1, n (%)			18 (60.0)	-	-	-	-	-
Type 2, n (%)			2 (6.7)	-	-	-	-	-
Not otherwise specified, n (%)			10 (33.3)	-	-	-	-	-
** Comorbidity, n (%)**								
None, n (%)			11 (36.7)	21 (70)	-	10.79	1	.21
ADHD, n (%)			11 (36.7)	6 (20)	-	-	-	-
OCD, n (%)			1 (3.3)	0 (0)	-	-	-	-
CD, n (%)			3 (10.0)	1 (3.3)	-	-	-	-
Anxiety disorder, n (%)			4 (13.3)	2 (6.7)	-	-	-	-
** Current Pharmacotherapy, n (%)**								
AD, n (%)			14 (46.7)	2 (6.7)	-	15.56	1	**<.01**
AP, n (%)			15 (50.0)	4 (13.3)	-	7.94	1	**<.01**
MS, n (%)			12 (40.0)	3 (10.0)	-	7.20	1	**<.01**
Stimulant, n (%)			7 (23.3)	6 (20.0)	-	1.0	1	.75
BDZ, n (%)			2 (6.7)	3 (10)	-	.22	1	.74

**Note**: Significant p-values (*p* < 0.05) are indicated in bold. **Abbreviations**: PBD, youth with pediatric bipolar disorder; BD-OFF, offspring of subject with bipolar disorder; HC, healthycontrols. CDRS, Children’s Depression Rating Scale. YMRS, Young Mania Rating Scale. ADHD, attention deficit hyperactivity disorder; OCD, obsessive-compulsive disorder; CD, conduct disorder. AD, antidepressant; AP, antipsychotic; MS, mood stabilizer; BDZ, benzodiazepine. Df, degrees of freedom; SD, standard deviation.

**Table 2:** CANTAB performances of 30 youth with PBD, 30 BD-OFF and 40 HC. Age, gender, IQ were used as covariates.

**Test**		**PBD ** **(*N* = 30)**	**BD-OFF ** **(*N* = 30)**	***HC*** **(*N* = 40)**	***f***	***Ancova*** ***df***	***p***	***Post-hoc***					
								**HC *vs*. ** **PBD**		**HC *vs*. BD-OFF**		**PBD *vs*. BD-OFF**	
								***p***	***d***	***p***	***d***	***p***	***d***
**AGN**													
RT-positive (sec.), mean ± SD		517.38 ± 107.97	524.66 ± 102.69	521.60 ± 84.16	1.46	5	.21	>.99	.04	>.99	.03	>.99	.06
RT-negative (sec.), mean ± SD		503.62 ± 104.81	527.86 ± 162.81	532.66 ± 95.12	.68	5	.65	.93	.29	1.00	.04	>.99	.17
CE-positive (n), mean ± SD		13.00 ± 6.18	10.30 ± 7.21	5.47 ± 4.72	6.31	5	**<.01**	**<.01**	1.36	**.02**	.79	.17	.40
CE-negative (n), mean ± SD		11.70 ± 6.61	9.13 ± 6.42	7.00 ± 6.08	2.99	5	.02	.01	.74	.74	.34	.29	.39
Omissions-positive (n), mean ± SD		13.43 ± 9.21	8.86 ± 6.01	5.75 ± 6.43	4.74	5	**<.01**	**<.01**	.96	.64	.49	**.03**	.58
Omissions-negative (n), mean ± SD		13.33 ± 9.48	9.73 ± 6.82	6.52 ± 6.75	3.91	5	<.01	<.01	.82	.36	.47	.18	.43
**CGT**													
Delay aversion, mean ± SD		.55 ± .40	.58 ± .042	.56 ± .04	1.43	5	.22	>.99	.03	>.99	.48	>.99	.10
Deliberation time (msec.), mean ± SD		347.50 ± 211.99	271.70 ± 217.95	277.60 ± 188.09	10.3	5	**<.01**	**.05**	.34	>.99	.02	**.04**	.35
Proportion bet, mean ± SD		.53 ± .02	.51 ± .03	.54 ± .02	.40	5	.85	>.99	.5	>.99	1.17	>.99	.78
Quality of decision making, mean ± SD		.89 ± .03	.84 ± .03	.84 ± .02	2.09	5	.07	.41	1.96	>.99	0	.56	1.66
Risk adjustment, mean ± SD		.39 ± .71	.62 ± .72	1.44 ± .63	.89	5	.49	.81	1.56	>.99	1.21	>.99	.32
Risk taking, mean ± SD		.55 ± .03	.56 ± .03	.58 ± .02	.49	5	.79	>.99	1.17	>.99	.78	>.99	.33
**SOC**													
Moves (n), mean ± SD		5.83 ± .28	6.30 ± .29	6.55 ± .25	7.32	5	**<.01**	.17	2.71	>.99	.92	.74	1.64
**MTS**													
% of correct choice, mean ± SD		96.07 ± .716	95.59 ± .74	96.94 ± .64	1.46	5	.21	>.99	1.28	.54	1.95	>.99	.65
**BLC**													
% of correct selection, mean ± SD		97.83 ± .50	98.53 ± .51	98.29 ± .44	.97	5	.44	>.99	.97	>.99	.50	.99	1.38

**Note**: Significant p-values (*p* < .05) are indicated in bold. **Abbreviations**: AGN, affective go/no-go; BLC, big/little circle; CANTAB, Cambridge Neuropsychological Test Automated Battery; CGT, Cambridge gambling task; CE, commission errors; MTS, match to sample visual search, SOC, stockings of Cambridge; RT, reaction times. PBD: youth with pediatric bipolar disorder; BD-OFF, offspring of subjects with bipolar disorder; HC, healthy controls. d, Cohen’s *d*; SD, standard deviation.

**Table 3:** Cerebellar total gray and white matter volumes (mm^3^) of 30 youth with PBD, 30 BD-OFF and 40 HC. Age, gender, IQ were used as covariates.

**Test**	-	**PBD ** **(N = 30)**	**BD-OFF ** **(N = 30)**	**HC ** **(N = 40)**	***ANCOVA***			***Post-Hoc***					
		**Mean (SD)**	**Mean (SD)**	**Mean (SD)**	**F**	**df**	***P***	**HC *vs.*** **PBD**		**HC *vs.*** **BD-OFF**		**PBD *vs.*** **BD-OFF**	
								***p***	***d***	***p***	***d***	***p***	***d***
Cerebellar gray matter (SD), mm^3^	Right	424.47 (60.65)	386.36 (52.47)	395.33 (45.53)	4.48	2	**.01**	**0.04**	.54	>.99	.18	.**03**	.67
	Left	425.97 (55.95)	386.98 (51.55)	395.67 (46.10)	5.12	2	**<.01**	.**02**	.59	>.99	.17	.**02**	.72
Cerebellar white matter (SD), mm^3^	Right	100.47 (19.83)	91.44 (14.24)	94.69 (13.31)	2.58	2	.08	.17	.34	>.99	.23	.14	.52
	Left	103.57 (19.31)	94.34 (14.48)	98.74 (16.23)	2.13	2	.12	.30	.27	>.99	.28	.19	.54

**Note**: ANCOVAs were performed on total cerebellar gray and white matter volumes corrected for intracranial volume (ICV). Significant p-values (*p* < .05) are indicated in bold. PBD, youth with pediatric bipolar disorder; BD-OFF, offspring of subjects with bipolar disorder; HC, healthy controls; d, Cohen’s *d*.

**Table 4:** F-values and r^2^ of univariate hierarchical linear regression of left and right cerebellum gray matter volumes and CANTAB tests scores in subjects with PBD, BD-OFF and HC. Results are corrected for age, gender, IQ.

	**CE-Positive**		**Omissions-Positive**		**Deliberation Time**	
**PBD**						
-	**F**	**R^2^**	**F**	**R^2^**	**F**	**R^2^**
Right cerebellar gray matter	**9.22**	**.13**	1.46	.05	.11	<.01
Left cerebellar gray matter	**9.33**	**.13**	.06	<.01	.10	<.01
**BD-OFF**						
-	**F**	**R^2^**	**F**	**R^2^**	**F**	**R^2^**
Right cerebellar gray matter	**6.34**	**.18**	2.01	.06	.17	.01
Left cerebellar gray matter	**6.35**	**.18**	2.03	.07	.23	.01
**HC**						
-	**F**	**R^2^**	**F**	**R^2^**	**F**	**R^2^**
Right cerebellar gray matter	3.20	.07	1.31	.03	.88	.02
Left cerebellar gray matter	4.37	.05	.82	.02	.88	.01

**Note**: Significant p-values (*p* < .05) are indicated in bold. **Abbreviations**: CE, commission errors, PBD, youth with pediatric bipolar disorder; BD-OFF, offspring of subjects with bipolar disorder; HC, healthy controls.

## Data Availability

Not applicable.

## LIST OF ABBREVIATIONS

AD: Antidepressant
ADHD: Attention Deficit Hyperactivity Disorder
AGN: Affective Go/No-go
AP: Antipsychotic
BD: Bipolar Disorder
BD-OFF: Offspring of Subject with Bipolar Disorder
BDZ: Benzodiazepines
BLC: Big/Little Circle
CANTAB: Cambridge Neuropsychological Test Automated Battery
CD: Conduct Disorder
CGT: Cambridge Gambling Task
HC: Healthy Controls
MS: Mood Stabilizer
MTS: Match to Sample Visual Search
OCD: Obsessive-compulsive Disorder
PBD: Pediatric Bipolar Disorder
SOC: Stockings of Cambridge
